# Identifying the Correlations Between the Semantics and the Phonology of American Sign Language and British Sign Language: A Vector Space Approach

**DOI:** 10.3389/fpsyg.2022.806471

**Published:** 2022-03-16

**Authors:** Aurora Martinez del Rio, Casey Ferrara, Sanghee J. Kim, Emre Hakgüder, Diane Brentari

**Affiliations:** ^1^Department of Linguistics, University of Chicago, Chicago, IL, United States; ^2^Department of Psychology, University of Chicago, Chicago, IL, United States

**Keywords:** sign language, ASL, BSL, iconicity, semantic modeling, vector space, computational methods

## Abstract

Over the history of research on sign languages, much scholarship has highlighted the pervasive presence of signs whose forms relate to their meaning in a non-arbitrary way. The presence of these forms suggests that sign language vocabularies are shaped, at least in part, by a pressure toward maintaining a link between form and meaning in wordforms. We use a vector space approach to test the ways this pressure might shape sign language vocabularies, examining how non-arbitrary forms are distributed within the lexicons of two unrelated sign languages. Vector space models situate the representations of words in a multi-dimensional space where the distance between words indexes their relatedness in meaning. Using phonological information from the vocabularies of American Sign Language (ASL) and British Sign Language (BSL), we tested whether increased similarity between the semantic representations of signs corresponds to increased phonological similarity. The results of the computational analysis showed a significant positive relationship between phonological form and semantic meaning for both sign languages, which was strongest when the sign language lexicons were organized into clusters of semantically related signs. The analysis also revealed variation in the strength of patterns across the form-meaning relationships seen between phonological parameters within each sign language, as well as between the two languages. This shows that while the connection between form and meaning is not entirely language specific, there are cross-linguistic differences in how these mappings are realized for signs in each language, suggesting that arbitrariness as well as cognitive or cultural influences may play a role in how these patterns are realized. The results of this analysis not only contribute to our understanding of the distribution of non-arbitrariness in sign language lexicons, but also demonstrate a new way that computational modeling can be harnessed in lexicon-wide investigations of sign languages.

## 1. Introduction

While wordforms are mapped to referents in a variety of ways, there is a growing body of evidence from spoken and signed languages demonstrating consistent trends in how the form of words can relate to their meaning. Some of these relationships are arbitrary, where the form of a lexical item has no connection to its referent other than through social convention, and some of these are non-arbitrary, where the meaning or function of an item can be predicted through some aspect of its form. For example, the meaning of a word like “tree” is not motivated by the letters or sounds making up its label. There is nothing about the sounds /t/ and /ɹ/ and /i/ that necessarily evoke “tree-ness,” and so the relationship between form and meaning in this case is considered arbitrary. In contrast, there are forms like “boom” and “roar,” which have a clear resemblance to their referent in their phonological form that can be said to exemplify a non-arbitrary relationship between form and meaning. Scholarship across languages and modalities has begun to test how these motivated relationships are distributed in the lexicon, and show patterns wherein there is a pervasive presence of words, across languages and modalities, whose meaning and whose phonological form are linked (Blasi et al., 2016; Dautriche et al., 2017b; Perlman et al., 2018). Here, building on this work, we use computational modeling to examine how these non-arbitrary form-meaning relationships are organized within the lexicons of two unrelated sign languages in order to better understand how a pressure toward non-arbitrary relationships between form and meaning might shape sign language lexicons.

Languages can exhibit multiple types of non-arbitrariness in their vocabularies, and do so in distinct ways across modalities. One form of non-arbitrariness expressed in language is **systematicity**,^1^ whereby patterns in how words are realized within a language correspond to word usage, and therefore meaning, in a statistical way (Dingemanse et al., 2015). Systematic cues in spoken languages, including vowel height, duration, stress, voicing, phonotactics, etc., have been found to correlate to syntactic, as well as semantic information (Kelly, 1992; Monaghan et al., 2007; Reilly et al., 2012). For example, in English disyllabics, stress often distinguishes verbs from nouns (ˈ**re**cord vs. reˈ**cord**, ˈ**per**mit vs perˈ**mit**). Systematicity is not limited to prosodic information, and can be found embedded in the form of the words themselves. In Semitic languages such as Arabic or Hebrew, many verbs and nouns are formed from a consonantal root, or a sequence of consonants that combine with vowels and non-root consonants to form semantically related terms. For example, in Arabic, the triconsonantal root “k - t- b” (ك ت ب) relates to the meaning “writing.” Words derived from this root are associated with writing along varying degrees of abstraction, such as كاتِب *k*ā*tib* (writer), كِتَاب *kit*ā*b* (book), مَكتَب *maktab* (office/desk), مَكتَبة *maktaba* (library), and many others (McCarthy, 1981). Systematic non-arbitrariness is also exemplified in phonesthemes (Firth, 1930), a type of non-morphemic sound-meaning pairing. We see this in the English onset *gl-* which often occurs with words relating to light, such as *glitter, glimmer, gleam, glisten, glow* (Bergen, 2004).

Systematic non-arbitrariness occurs in sign languages as well, as is the case in sign families. Sign families refer to “groups of signs each with a formational similarity and a corresponding meaning similarity” (Frishberg and Gough, 2000). In ASL, the signs mock, ironic, and stuck-up form such a family, each articulated with the 1-I or “horns” handshape (the index and pinkie finger extended). All three signs have some implied negative meaning and conform to a pattern in ASL in which signs produced with the 1-I handshape often have such connotations; however, this correspondence is likely to be language-specific as it is not derived from any transparent visual resemblance^2^.

A second form of non-arbitrariness is **iconicity**^3^. Iconicity here refers to a motivated relationship between form and meaning by way of perceptuomotor resemblance-based analogies (Frishberg, 1975; Dingemanse et al., 2015). Historically, iconicity was considered exceedingly rare in spoken languages, and exemplified only in onomatopoeia, where the forms of words have a direct resemblance to their referent *via* their phonology. However, more recent work has found iconicity to be both fundamental and pervasive in human communication (Perniss et al., 2010; Vigliocco et al., 2014; Akita and Dingemanse, 2019). This becomes particularly clear when looking beyond Indo-European languages, where many spoken languages have been found to possess rich inventories of words known as ideophones, which iconically represent a multitude of sensory impressions such as movements, textures, sounds, visual patterns, even cognitive states (Diffloth, 1972, 1979; Kita, 1997).

Sign languages provide further evidence of the pervasiveness of iconicity. Because they exist in the visual modality, sign languages bring with them new affordances. In much the same way that iconicity in spoken languages often makes use of sound-based symbolism (e.g., onomatopoeia), communicating in the visual modality allows for the iconic representation of visual information more readily. Because of this increased ease of visual-to-visual mapping, as well as the prevalence of visual information in everyday communication, it is perhaps unsurprising that sign languages are considered to be more iconic than spoken languages (Klima and Bellugi, 1979; Taub, 1997; Meier, 2009; Perlman et al., 2018)^4^. Although iconicity has been noted to be more wide-spread in the vocabularies in sign languages, it is important to note that most signs in the lexicon are not highly iconic (see Caselli et al. 2017 and Sehyr et al. 2021 for a review of the distribution of iconicity in ASL).

**Figure 1:**
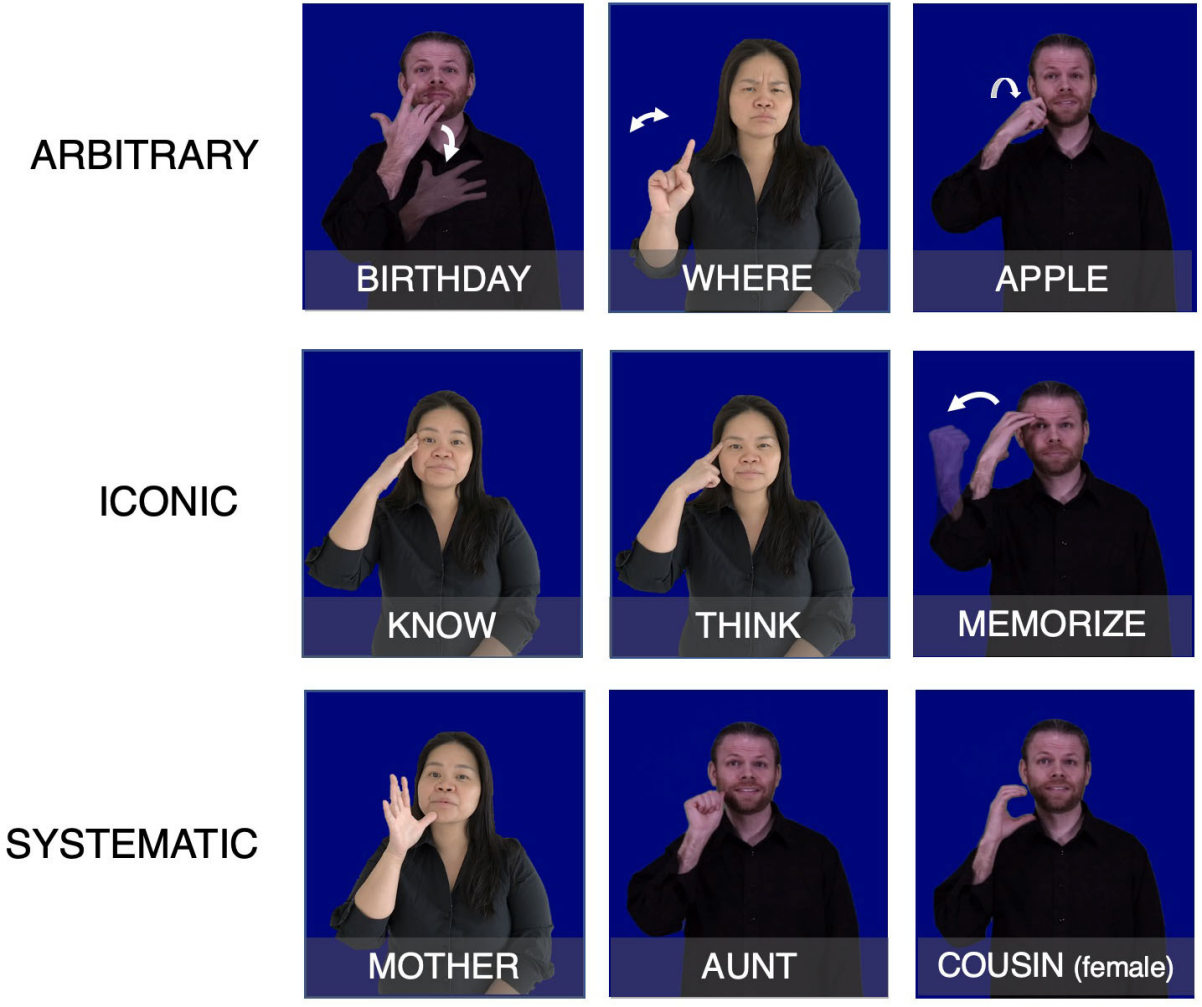
Examples of arbitrary **(top row)**, iconic **(middle row)**, and systematic **(bottom row)** signs in ASL. The verbs of cognition in the middle row are all articulated on the head, which indicates their relationship to the brain through iconicity. The kinship signs in the bottom row are all articulated near the chin, which is conventionally associated with feminine roles among ASL kinship signs.

Words can also combine elements of both systematicity and iconicity (as well as arbitrariness) together in a wordform. For example, in ASL, many signs relating to feelings or emotional states are articulated on or near the chest. This overlap in location is iconically motivated, based on these concepts relating to one's heart. Many of these sign are also articulated with an open-8 handshape (hand is open with the middle finger bent at the first knuckle). The overlap in handshape here is based on an ASL convention whereby this handshape connotes an association with feelings, and is systematic rather than iconic. Figure 1 shows examples of arbitrary, systematic, and iconic signs in American Sign Language (ASL).

Together, this points to how spoken and signed languages make use of non-arbitrariness in the mapping of different wordforms to their referents, and demonstrates that non-arbitrariness may be derived iconically, systematically, or both to various degrees. While the pervasiveness of non-arbitrary form-meaning mappings can be seen cross-linguistically, many wordforms also retain completely arbitrary relations to their referents, and so a question remains regarding not only how, but also why non-arbitrariness is distributed across linguistic systems.

One factor influencing how form is mapped to meaning is the communicative and cognitive pressures^5^ that shape the organization of the lexicon. One such pressure is toward a low degree of similarity or overlap between wordforms. There is evidence that phonological systems are shaped to be maximally distinctive while also cost-effective (see *Feature Economy* in Clements 2003). In other words, in examining how phonological features are combined across a lexicon, languages tend to combine the features available to them in as many ways as possible to maximize the number of distinct forms. Referred to as “dispersion” (Flemming, 2002, 2017; Dautriche et al., 2017a), this tendency appears to maximize perceptual clarity to reduce uncertainty for the perceiver. This is supported by evidence for the negative effect of neighborhood density on spoken word recognition, where words from high density neighborhoods tend to be processed more slowly and less accurately (Luce and Pisoni, 1998). Additionally, research suggests that young children struggle to assign distinct meaning to novel wordforms when those wordforms have a high degree of phonological overlap to ones already in their lexicon (Dautriche et al., 2015), suggesting that a lack of dispersion among lexical items could interfere with early vocabulary building.

There is also evidence for a contrasting pressure toward similarity among wordforms. While dispersion allows for high perceptual clarity on the part of the perceiver, there are additional functional advantages for “clumsiness” (Monaghan et al., 2011; Dautriche et al., 2017a), or the tendency toward higher phonological overlap between the wordforms in a lexicon. Words with many phonological neighbors show improved recall over more distinct words, and show facilitated production as evidenced by lower speech error rates (Vitevitch and Sommers, 2003; Stemberger, 2004; Vitevitch et al., 2012). There is evidence that this pressure shapes the distribution of phonological forms across lexicons, as demonstrated by Dautriche et al. (2017a)'s analysis showing that in spoken languages, lexicons are organized into phonologically similar clumps.

These contrasting pressures, toward increased dispersion on the one hand and increased similarity on the other, crucially interact with the presence of non-arbitrary form-meaning mappings in the lexicon. Evidence of the impact of this pressure toward similarity on the organization of the lexicon, and its inter-action with non-arbitrariness, can be seen in findings showing that phonologically similar words tend to be semantically similar within and across languages (Monaghan et al., 2014; Blasi et al., 2016; Dautriche et al., 2017b). For example, in one computational analysis comparing the semantic and phonological distance between words in 100 spoken languages, Dautriche et al. (2017b) found a weak trend where more semantically similar word pairs were also more phonologically similar. Likewise, in Blasi et al. (2016), there was a consistent presence of non-arbitrary sound-meaning associations in the sounds used for a subset of basic vocabulary items across thousands of unrelated spoken languages. We can also see this relationship between converging form and meaning in the presence of non-arbitrariness in sign languages, as shown by groups of semantically related terms that have shared features that are represented iconically, such as the location of the signs know, think, memorize in ASL (see Figure 1). Groupings of wordforms like this have been investigated in studies on both spoken and sign languages that have shown that denser semantic neighborhoods exhibit greater degrees of iconicity than more sparsely distributed neighborhoods (Sidhu and Pexman, 2018; Thompson et al., 2020a), suggesting further areas where aspects of meaning and form may come together. We aim to investigate the pervasiveness of non-arbitrariness in sign languages and their impact on the organization of the lexicon to provide further insight into how these contrasting pressures might shape the lexicons of sign languages.

**Figure 2:**
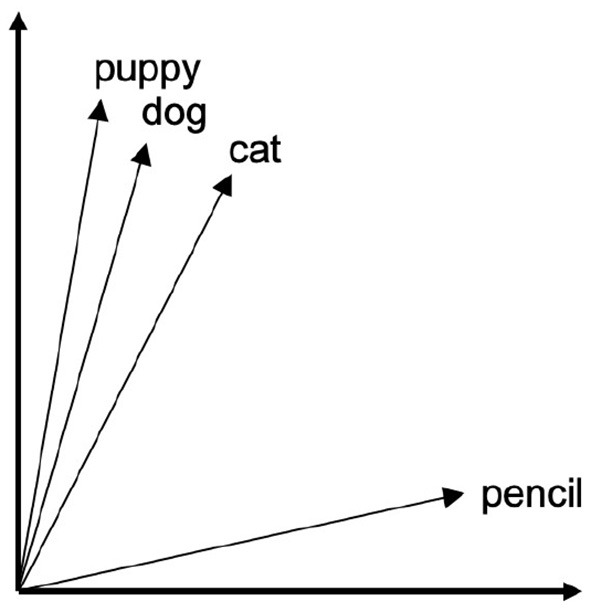
Hypothetical vector space for four English words.

However, understanding the distribution of these form-meaning relationships across an entire lexicon requires a way of defining a word's meaning such that it can be abstracted and compared to other word meanings in a quantitative way. One way that researchers have endeavored to do this is based on the distributional hypothesis (Harris, 1954; Firth, 1957; Turney and Pantel, 2010). Summarized by Firth (1957) as “know[ing] a word by the company it keeps,” this principle proposes that the meaning of a word can be derived from the contexts across which it is distributed, such that words which occur in similar contexts are likely similar in meaning. This forms the basis of distributional models, such as vector space models (VSMs), where words are represented as vectors situated in a “semantic space.” In these models, a word's proximity to other words indicates their relatedness in meaning. Similarity is then computed by deriving the cosine of the angle between the two vectors (for review, see Erk 2012). This concept is exemplified in Figure 2, which shows semantic similarity as represented through proximity in a simplified vector space of four English words^6^.

**Figure 3:**
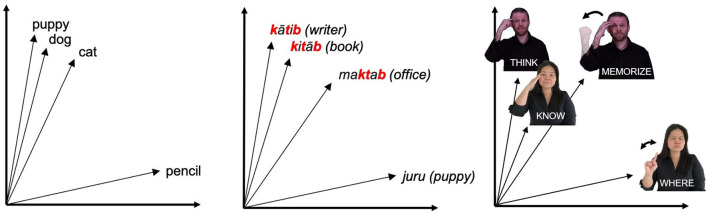
Example English and Arabic words and ASL signs represented in vector spaces.

Operationalizing meaning in this way provides an intuitive notion of distance, and allows us to compute similarity between words algebraically. This advantage has made VSMs a powerful and widely-used tool in computational linguistics (see Clark 2015 for review). VSMs have been shown by Thompson et al. (2020a) to be an effective tool in studying systematic correspondences in meaning between signs for sign languages, as demonstrated in an analysis wherein VSM models were used to test the relationship between semantic density and the distribution of iconicity in the lexicon of ASL. For the present analysis, vector space models can also serve as a useful tool for exploring how non-arbitrariness may interact with phonological form in the organization of the lexicon, as relatedness in meaning can be quantified using these models and compared to relatedness in form. Figure 3 shows an example of this through a comparison of three hypothetical vector spaces in English, Arabic, and ASL.

For each graph, some of the words form a cluster due to their close semantic relationship. Note that in the leftmost vector space (English), the clustered words are phonologically dissimilar while in the center and rightmost vector spaces (Arabic and ASL, respectively), the clustered words share certain phonological features with each other. In the case of Arabic, this phonological overlap is due to the shared root k-t-b (root consonants noted in red), which in Arabic indicates that all of these words have some relationship to writing. In the ASL example, the shared phonological feature of these signs is their location on the head, which instead has an iconic motivation, as these signs all relate to mental states. In instances of non-arbitrariness in sign language, such as that exemplified above, we predict that phonological information should be evident in the organization of semantics, whether it be systematically or iconically derived. Sign languages in particular are a valuable area in which to apply this vector space approach, due to the pervasiveness of visual iconicity relative to many spoken languages, and computational approaches to the modeling of sign languages are an essential, yet under-explored area in the field.

We hypothesize that as a general property of sign language lexicons, there will be a positive correspondence between semantic similarity and phonological similarity, and test this hypothesis using a computational approach for two unrelated languages: ASL and BSL. For each sign language, we examine the relationship between the phonological overlap of signs and their semantic similarity as determined by their proximity to one another within the vector space model. We take two approaches to testing this hypothesis, first testing for correlations between phonological similarity and semantic similarity between all possible pairs of signs in our corpora (pairwise comparison), and then again within the boundaries of semantically similar clusters of signs (clustering analysis). Our pairwise analysis follows analyses of spoken languages like Dautriche et al. (2017b) and Blasi et al. (2016), that show a relationship between phonological and semantic similarity in the lexicons of many spoken languages, and allows us to test whether there is a correspondence between semantic and phonological similarity across the lexicons for two unrelated sign languages. The clustering analysis then takes this a step further to test whether we see systematic patterns in the grouping of phonological and semantic information within the lexicons of ASL and BSL.

## 2. Materials and Methods

### 2.1. Phonological Data

The phonological information about signs, used to determine phonological similarity, comes from annotated databases of signs from ASL and BSL. The data used for the analysis encompass two annotated datasets of signs that are drawn from existing lexical databases: *The Gallaudet Dictionary of American Sign Language* (Valli, 2006) and BSL SignBank (Fenlon et al., 2014). The American Sign Language dataset comprises annotations of videos of lexical signs from *The Gallaudet Dictionary of American Sign Language*, with the project dataset including 2,698 videos of lexical signs^7^ from the video dictionary. The British Sign Language dataset comprises annotations of videos of lexical signs available in the public view web dictionary of the BSL SignBank (Fenlon et al., 2014), encompassing a total of 2,337 unique video entries of lexical items. For signs that have multiple variants, or entries, in the datasets we only use the first listed variant for the present analysis to avoid skewing the data. For example, in the BSL Signbank, there are 15 different entries for the sign mauve, but only the first listed was included in the analysis. After removing the duplicates, we were left with 2,335 unique ASL lexical entries, and 1,630 unique BSL lexical entries.

The signs included in the project dataset each received an annotation for their phonological properties within the handshape, location, and movement parameters. The project datasets were annotated by research assistants in the *Sign Language Linguistics Laboratory* at the University of Chicago. Handshape was annotated using the system developed in Eccarius and Brentari (2008). This annotation system uses a combination of letters and numbers to represent the distinctive features that comprise each handshape as represented in the Prosodic Model (Brentari, 1998, 2019). This encompasses not only distinctions in selected finger groupings, but also in joint configuration and the position of the thumb. The location coding system captures specific distinctions in minor location within five major location zones (the head, body, arm, hand, and neutral space). For the handshape and location annotation schema, there are separate annotations for the dominant and non-dominant hands, as well as for their specifications at the beginning and the end of each sign. The movement annotation system is based on meaningful contrasts in the movement parameter as outlined in Brentari (1998). The movement annotation system encompasses distinctions within the categories of local movement, path movement, axis, and the behavior of the non-dominant hand with respect to the dominant hand.

### 2.2. Similarity Measurements: Semantic and Phonological Similarity

Both semantic and phonological similarity were determined through computational modeling by calculating the semantic and phonological distance between signs. More specifically, within these models, the phonological and semantic relationships between signs are represented through multi-dimensional vector spaces, where similarity is determined by proximity within these spaces. As a way to quantify and compare similarity between words, we use cosine similarity to measure the distance between two vectors in the embedding space.

Beginning with **semantic similarity**, in computationally modeling word meanings, the meanings of words are represented by a vector in a high-dimensional semantic space. For our analysis, we used pre-trained English word embeddings to measure semantic similarity between pairs of signs, because the lack of a large enough sign language corpus to train a reliable vector space necessitated that we use word embeddings trained on spoken English corpora. We made this decision following Thompson et al. (2020b)'s cross-linguistic study which shows that cultural proximity is an indicator of semantic alignment between languages. We used the embedding vectors from the *Global Vectors for Word Representation* (GloVe) algorithm (Pennington et al., 2014)^8^. Only signs with corresponding semantic vector representations in the GloVe vectors were included in the analysis. This left us with 1946 signs for ASL and 1480 signs for BSL. The two datasets overlapped partially in meaning, with 590 signs overlapping in meaning between the two datasets. This difference may be due in part to the differing nature of the datasets, where one is a dictionary and the other a signbank, or simply due to different decisions made in compiling the two lexical databases.

While semantic similarity was calculated using pre-trained word embeddings, **phonological similarity** was calculated from a vectorized phonological space that was constructed using the phonological datasets. This was achieved by vectorizing the phonological specifications for each of the signs in each dataset, using one-hot-encoding to assign each sign a phonological vector. We used the annotated labels of the phonological specifications for each of the phonological parameters—handshape, location, and movement—for the one-hot-encoding process.

Each sign was represented as a vector with dummy variables (0 or 1) after applying one-hot-encoding to the phonology data. We then applied dimensionality reduction by means of truncated singular value decomposition (SVD) to the one-hot encoded data, a commonly used computational method to transform data for efficient computation and capturing generalizations^9^. We approximated the phonological similarity through the phonological distance between the vectorized representations of the signs in our dataset. Phonological similarity, as with semantic similarity, was obtained by calculating the cosine similarity between the phonological vector representations for each sign pair.

This approach to calculating phonological similarity was chosen because it provides a similarity metric that is comparable to the vector-based metric used to determine semantic similarity. While this is the approach chosen for this analysis, it is worth noting that there exist other measures of phonological similarity that might yield different results. For example, there are finer grained methods of calculating phonological similarity, such as the handshape similarity metric elaborated in Keane et al. (2017). Because the current method of calculating phonological similarity does not capture finer grained distinctions between phonological specifications, such as, for example, gradient differences in joint flexion, we expect that methods incorporating these distinctions would potentially show stronger relationships between phonologically similar signs, but we leave this to future work.

## 3. Results

We take two perspectives on analyzing the data: (i) a pairwise comparison and (ii) a clustering analysis. The same approach to calculating phonological and semantic similarity was used for both analyses. In the first of these, the **pairwise comparison**, we look at the relationship between semantics and phonology across the lexicon as a whole, by finding the phonological similarity and the semantic similarity between all possible pairs of signs in the available vocabularies. In the **clustering analysis**, we first break the vocabulary into groups of semantically similar signs and then run our similarity metrics within the boundaries of these semantic clusters. The two analyses were chosen to test different generalizations about how the phonology of sign languages might be mapped onto meaning components.

### 3.1. Pairwise Comparison

When we look at general trends in the relationship between semantic and phonological similarity for the vocabularies of ASL and BSL, we predict a positive relationship between the two. This prediction is based on trends from spoken languages where there is a present, albeit weak, association between form and meaning, as well as due to the noted pervasiveness of form-meaning mappings in sign language. We test this by finding the semantic and phonological similarity between each pair of signs for all of the unordered pairs of signs in each dataset. For instance, the filtered ASL vocabulary of size 1946 has 1892485 unordered sign pairs. For every one of these pairs, we find the phonological and the semantic similarity between the two members of the sign pair.

**Figure 4:**
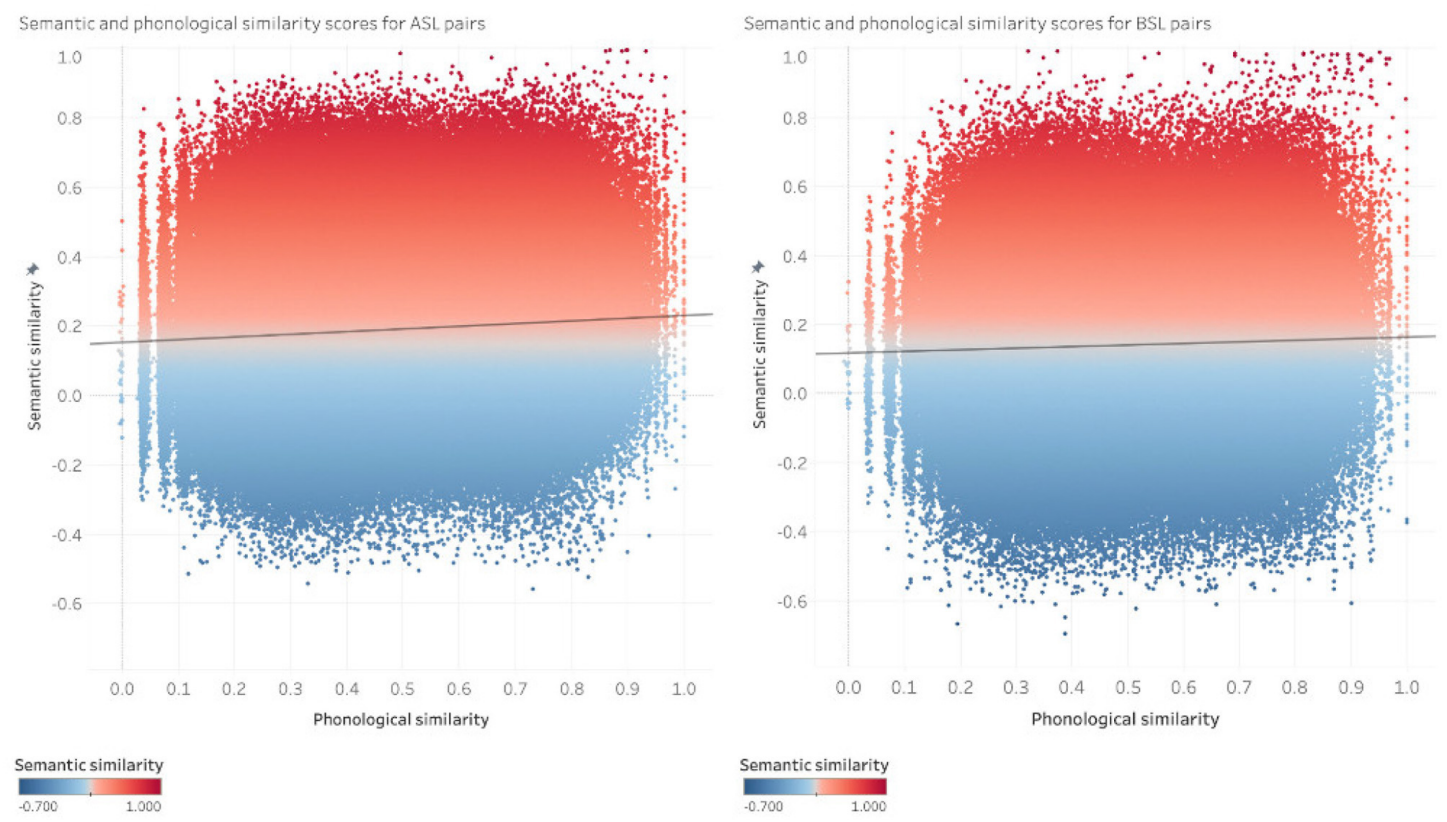
Pairwise comparisons in 100-dimensional GloVe vectors in ASL **(left)** and BSL **(right)**. Each point is a pair of signs *[s1, s2]*; *x*-axes show phonological similarity, *y*-axes show semantic similarity.

We analyzed the relationship between the semantic and phonological similarity of pairs of signs in the ASL and BSL datasets using a Pearson correlation. We report the results for the correlation analysis across sign pairs in both the ASL and the BSL datasets. Results are reported for the 100 dimensional semantic space, as each of the semantic spaces tested (100, 200, and 300 dimensions) show negligible differences between them in the pairwise analysis. This relationship between the semantic and phonological similarity for every pair of signs in the ASL dataset and BSL datasets is shown in Figure 4.

Our results show a weak but significant positive relationship between semantic and phonological similarity for both of the ASL or the BSL vocabularies (ASL: r(1892483) = 0.074, *p*< *0.001*; BSL: r(1094458) = 0.044, *p*<*0.001*). The correspondence between semantic and phonological similarity is evidenced by pairs of signs in the dataset like [thursday, tuesday] in ASL and [four, three] in BSL, which are semantically highly related while also phonologically very similar. Although there is a positive relationship between semantic and phonological similarity between the sign pairs, the correlation coefficients for both languages are quite low (all *r* < 0.1), showing that while there is a positive correlation between phonological and semantic similarity in the lexicons for both languages, this is not a strong trend. This weak trend is exemplified by the highly dispersed distribution of semantic and phonological similarity between sign pairs across the vocabularies of ASL and BSL. As seen in Figure 4, for ASL and BSL pairs of signs are distributed such that there are not only pairs of signs that show a correspondence between phonological and semantic similarity, but there are also a considerable number of pairs that are semantically similar and phonologically quite distinct, like [grass, flower] in ASL and [banana, apple] in BSL, as well as pairs of signs that are phonologically similar and semantically distinct, like [lotion, senior] in ASL and [addition, vomit] in BSL.

### 3.2. Clustering Analysis

In the previous section, we showed that pairwise comparisons between signs do not reveal strong correlations between semantic similarity and phonological similarity. Here, we take a different perspective from the pairwise comparison and report our findings on the dataset when it is clustered into sets of semantically related signs, with the prediction that we will see a stronger positive correspondence between phonological and semantic similarity within these groupings. This approach is motivated by evidence that lexicons not only exhibit some degree of clustering in their phonological material (Dautriche et al., 2017a), but also by evidence that areas of more densely clustered semantic space tend to be more iconic (Sidhu and Pexman, 2018; Thompson et al., 2020a).

We use a hierarchical clustering algorithm in this analysis. Hierarchical clustering is a statistical clustering technique which creates a tree hierarchy of inter-connected clusters instead of creating independent ones. We take the bottom-up (agglomerative) approach where each point in the high-dimensional space starts out as its own cluster and merges with other nodes or clusters hierarchically with respect to their Euclidean distance from one another. We use Ward's method (Ward, 1963) where the decision to merge clusters at each step is made based on the optimal value of a loss function—this method minimizes the within-cluster variance.

**Figure 5:**
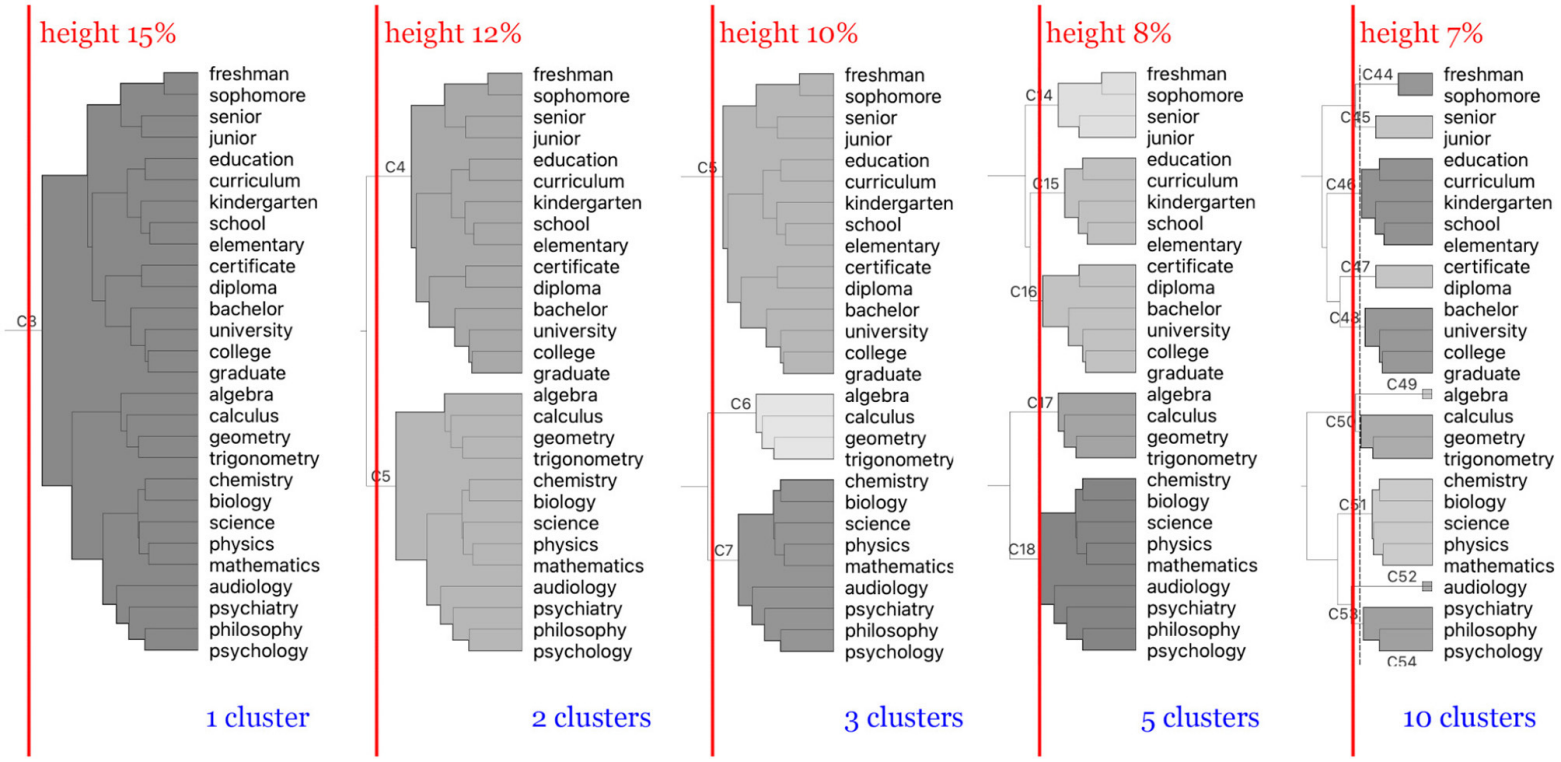
Different groupings by pruning height of signs in the ASL dataset with respect to different pruning heights indicated with the red lines. As the pruning height decreases from left to right, the number of clusters increases. Lower heights prune the hierarchical tree at junctions closer to the terminal leaf nodes; hence, a larger number of resulting clusters with fewer members.

Hierarchical clustering creates a tree of inter-connected branches and leaves where the final clusters are determined by a **pruning height**. Figure 5 shows how different values of pruning heights for ASL form different sized clusters of signs grouped by their semantic proximity to one another. In this study, we investigated the data clustered using 100 different pruning heights (height of 0% through 100% at 1% intervals). Different height values produce vastly different groupings in the hierarchical tree with smaller values producing a larger number of clusters composed of fewer members and larger values producing a smaller number of clusters with larger populations.

After the clustering step, we took the same steps as we did with the pairwise comparison method. We identified the unordered pairs of signs in each cluster and measured the semantic and phonological similarity within each pair; however, the pairing step did not cross cluster boundaries. We evaluated the quality of our clustering algorithm across variable dimensions and heights using silhouette scores. **Silhouette scores** are commonly used as a measure of consistency within clusters and distinctiveness from other clusters, providing a metric of how well the models are grouping the semantic space. Higher silhouette scores indicate that data points within a cluster are better matched to each other, and the cluster is dense and more easily separable from other clusters.

**Figure 6:**
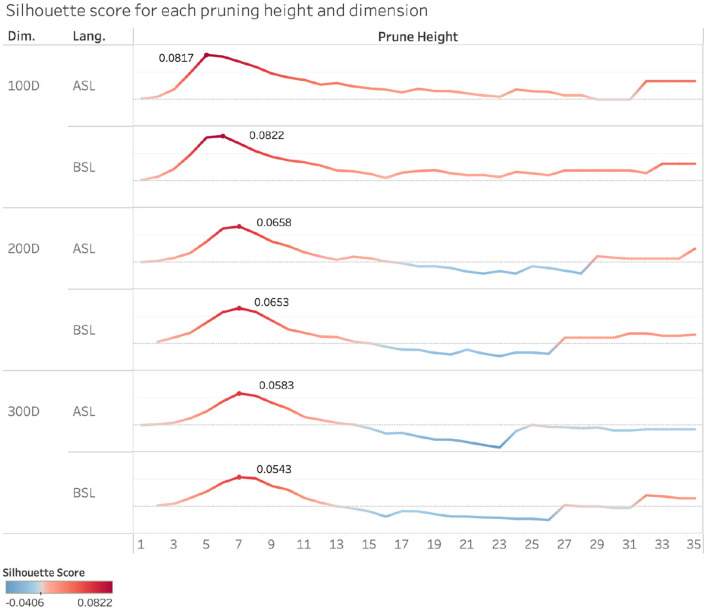
Silhouette scores in ASL and BSL across semantic spaces (100-, 200-, and 300-dimensional semantic space) and pruning heights. (1–35%). A score of 0 indicates there are multiple data points that overlap in clusters. A scores of -1 indicates that data points are assigned to incorrect clusters.

Figure 6 illustrates the silhouette scores for each pruning height and dimension. Analysis of the scores reveals a consistent pattern across the different numbers of dimensions and across both sign languages, where the silhouette scores rise sharply as pruning height begins to increase, peak within a pruning height range between 5 and 10%, and then drop back down as the pruning height continues to increase, eventually appearing to plateau. This indicates that our sign clusters are the most well-defined within this 5 and 10% range in each of these vector spaces, in particular for the 100 dimension space, where these peaks were the highest in both ASL and BSL. It is at these pruning heights and dimensionality of the vector space that the semantic space is most well organized.

**Figure 7:**
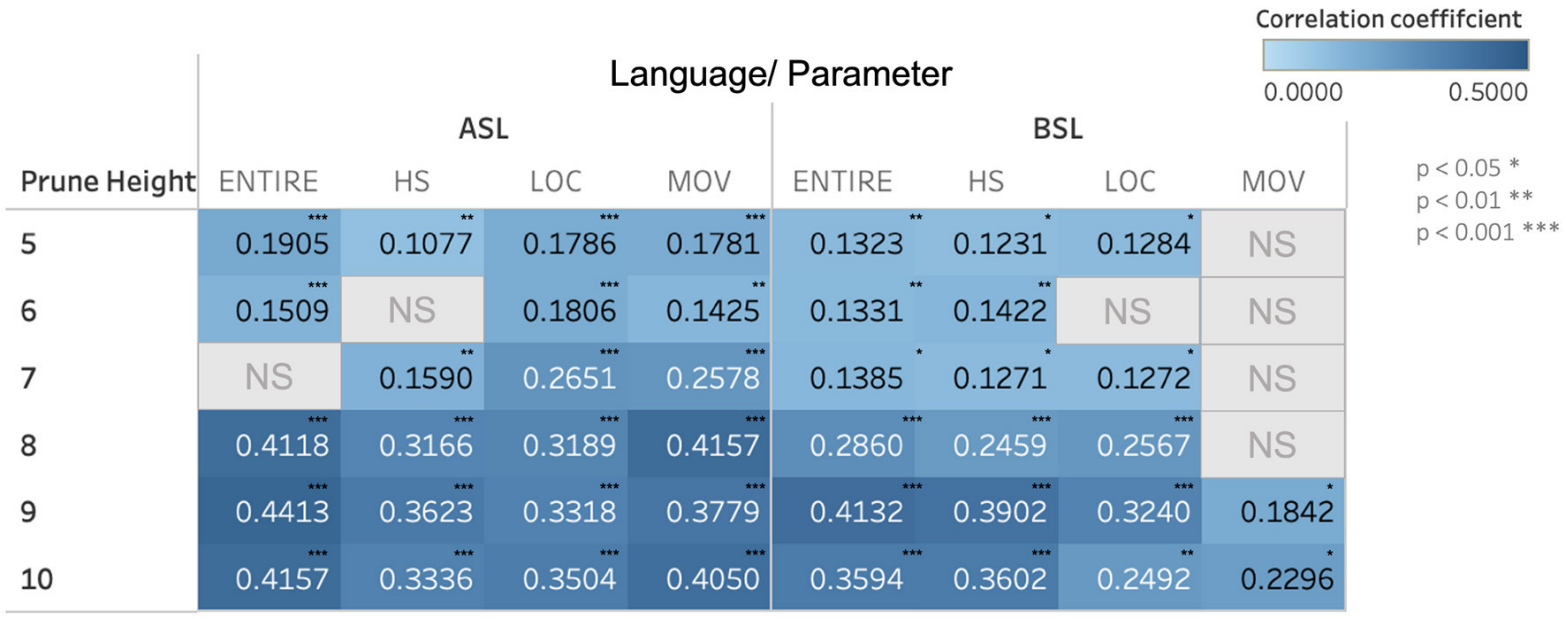
Pearson's correlation coefficients between semantic and phonological similarity for the 100-dimension semantic vector space for ASL and BSL. Correlation coefficients are listed for all of the parameters combined (“entire”) and each of the parameters individually (“HS” = handshape, “LOC” = location, and “MOV” = movement). Relationships that were not significant are marked with “NS.”

When we examine the correspondence between semantics and phonology within clusters across these pruning heights, we find a range of small to moderately large positive correlations, the majority of which are significant. We calculated the strength of the relationship between semantic and phonological similarity in all sign pairs by using Pearson's correlations. Figure 7 shows the correlation coefficients for the 100-dimension semantic vector space in ASL and BSL from pruning heights 5–10 for each phonological parameter.

As shown in Figure 7, as pruning height increases, the strength of the relationship between semantic and phonological similarity increases. This trend is broadly applicable to both languages and all three phonological parameters we investigated. However, the correlation strength between semantic and phonological similarity is different between ASL and BSL. In BSL, the correlations are consistently weaker than in ASL, as evidenced by the lower correlation coefficients.

The languages also exhibit different trends with regard to which phonological parameters (handshape, location, and movement) show stronger correlations with semantic similarity. For example, along the movement parameter in ASL, there is a consistent positive correlation between phonological and semantic similarity that increases along with increased pruning height. In contrast, for many of the pruning heights examined for BSL, this relationship was either not significant, or was lower in strength than for ASL.

## 4. Discussion

In this investigation, we used a vector space approach to investigate and quantify the form meaning relationships within the vocabularies of two unrelated sign languages. This method of modeling the semantic and phonological spaces allows us to probe this relationship quantitatively. The first analytic approach taken tested the relationship between the semantic and phonological similarity of *all* of the sign pairs in the BSL and ASL lexicons, while the second of these analyzed this relationship within the bounds of semantically clustered groups of signs in the ASL and BSL lexicons.

### 4.1. Discussion of the Pairwise Comparison

For the pairwise comparison, our results showed a significant, but weak correlation between the semantic and phonological similarity for pairs of signs across the lexicons of ASL and BSL. These results align with previous studies on spoken languages, for example in those of Dautriche et al. (2017b) and Blasi et al. (2016), that show some degree of systematic patterning between the meaning and form of vocabulary items for spoken languages. The positive relationship seen here may stem not only from the affordances of signed languages, which can leverage the visual modality to represent particular systematicities between form and meaning, but also from other wider pressures. For example, non-arbitrariness has been shown to have a positive contribution to the learnability of words (Imai et al., 2008; Monaghan et al., 2011), which might then contribute to a pressure toward retaining non-arbitrary forms.

The strength of the correlation found in the analysis can also be explained in part by the multiple competing forces that contribute to the phonological organization of the lexicon. As discussed previously, phonological systems are shaped in part by a pressure to be maximally distinctive and to combine all the features available to them in a cost-effective way (Clements, 2003). If we expected that the only factor driving phonological form lay in the semantics of signs, we would bypass this cost-effective property of phonology. Following from this, when we think of the lexicon as a whole, sign pairs that are distant in their semantics but similar in their phonological form are expected, due to the maximization of the combinatorial possibilities of the phonological features available.

Lexical items that are more similar to one another may also be more confusable, leading to additional pressures on forms to be more phonologically dissimilar from one another (Dautriche et al., 2017b). There is also evidence from ASL that signs in denser phonological neighborhoods are recognized more slowly for lower frequency signs (Caselli et al., 2021), and so phonological distinctiveness may play a role in facilitating sign recognition. Together these provide evidence of some pressure toward distinctiviness, which might be contributing to the weak trend seen in this analysis.

In a similar vein, although there are signs that are broadly conceptually similar, there are reasons that lie within their visual properties and iconic affordances that would lead us to expect them not to share phonological properties. For example, consider the broad semantic category of animals. While we might expect some animals to share some iconic properties that might be reflected in their signs, such as body parts (beaks, ears, or tails) or aspects of how they are handled by humans, these properties would not be shared across the entire semantic category of animals. In fact, signs that mapped their phonological form to some of these visual properties, such as beaks and tails, would in fact be more dissimilar from one another due to the differing properties of their referents. On the other hand, within a narrower semantic category, such as that of birds, there are more shared visual features between referents that might result in increased similarity in their phonological form. In this way, sign pairs within more narrowly delineated semantic categories would be more likely to share phonological properties, but it would be unexpected for them to share properties across broader semantic categories. For this reason, we don't necessarily expect a linear relationship between similarity in meaning and in form across the broader lexicon. However, this leads to the prediction that signs grouped at particular levels might still show systematic relationships between their phonological form and their meaning.

### 4.2. Discussion of the Clustering Analysis

This leads us to our hierarchical clustering analysis, which showed that when signs in ASL and BSL are organized into semantic clusters, we find a systematic correspondence between semantics and phonology among sign pairs. The analysis of the silhouette coefficients demonstrated that the lexicon was most semantically well-organized between pruning heights of approximately 5 and 10% (as seen in Figure 6). Examining this alongside our correlation analysis, there was a significant positive correlation between semantic and phonological similarity at these pruning heights. This means that when the sign language vocabularies are organized at these levels, that is, into categories that are neither too broad nor too narrow, we see a relationship between semantic and phonological organization.

The findings of the clustering analysis provide further insight into the distribution of non-arbitrary relationships in the lexicons of sign languages by revealing a textured vocabulary where form and meaning are grouped, or clustered, together in systematic ways. The lexicon of ASL has been shown previously to include multiple clusters of highly iconic, semantically related signs (Thompson et al., 2020a). Following from this, if these clusters also share some degree of phonological similarity, this may contribute to the positive correlation between phonological and semantic similarity within the clusters in the present analysis. The relationships within these clusters also aligns with accounts that suggest a pressure toward a “clumpier” lexicon (Dautriche et al., 2017a), as in this case, the distribution of phonological material of signs is drawn closely together into clumps within the bounds of particular semantic groupings.

Although both ASL and BSL both showed a correspondence between phonological and semantic similarity within clusters, as noted in the analysis of the correlation patterns for ASL and BSL, differing trends appeared both between the two sign languages analyzed and between the correlation strengths for the different phonological parameters, as can be seen in Figure 7). As one example, the strength of the correlations for the movement parameter differed between ASL and BSL. In ASL, the movement parameter had stronger correlation coefficients at most pruning heights when compared to handshape and location. The opposite was true for BSL, where the correlation coefficient for the movement parameter was either not as strong as the other parameters or did not show a significant relationship. This suggests that within each language there may be differing tendencies in how meaning is mapped onto particular parts of the phonology in forming lexical items. One possible explanation for this pattern is that, for ASL, movement may be employed in more systematic ways to convey aspects of meaning, iconic or otherwise, while this may be relegated to the other parameters for BSL. For example, in ASL, the set of signs science, chemistry, and biology are all articulated with same circular path movement. ASL may employ the movement parameter to a greater degree than BSL to connect signs in semantically related schema like the one exemplified by this set of signs.

Another notable tendency in the clustering correlation analysis was in the differing strengths of the correlations for ASL and BSL as a whole. More specifically, across the pruning heights examined, ASL tended to have stronger correlations, evidenced by higher correlation coefficients between semantic and phonological similarity than BSL. Possible explanations for these differences can be drawn from the histories of the languages themselves and from the composition of the datasets used for the analysis. One explanation might lie in the relative age of the two languages examined in the study. ASL is a fairly young language, with its history stretching back roughly 200 years, while BSL is considerably older than ASL. One potential explanation for the weaker correlation in BSL is that signs that may have been iconic, over time, changed in their form enough that the strength of the association between form and meaning decreased. This would be in line with trends shown in previous research wherein phonological forms become less iconic and more abstract over time (Frishberg, 1975; Sandler et al., 2011). However, recent scholarship on iconicity and language change in spoken English also suggests that iconic forms are more stable and less likely to change over time (Monaghan and Roberts, 2021) and so the appealing to the age of ASL and BSL may not provide a comprehensive explanation of the patterns seen here. Another potential explanation is a methodological one. The datasets used in the analysis for ASL and BSL were of different sizes. The smaller size of the dataset used for BSL could provide one explanation for the difference in effect size seen for the two languages. Further research, with datasets of equal sizes, will provide more insight into any potential differences between the semantic and phonological organization of these two languages, as the trends seen here could be explained as reflecting cross-linguistic differences or could be a result of the project methodology. Because differences in the results could be due to the different vocabularies and dataset sizes used for each language, these comparisons and interpretations are all drawn tentatively.

### 4.3. Individual Clusters, Iconicity, and Systematicity

When considering what types of semantic features will organize clusters of non-arbitrary forms, there are likely multiple influences at play. One constraint influencing the presence of iconicity is the kinds of correspondences possible in a given part of the vocabulary. For example, meanings related to magnitude, intensity, or timing allow for iconic representations fairly well through characteristics such as word-length, volume, repetition, or speed of articulation. However, for more abstract concepts, there exists little opportunity for any form of imitative representation. Likewise, the possibilities for iconicity vary with the affordances of a given modality. Meanings related to sounds, or referents for which sound is an identifying feature, can be readily represented iconically in a spoken language, while visual and spatial information lends itself to iconic representation to a greater degree in a signed language (Dingemanse et al., 2015). These factors interact with pressures toward distinctiveness and similarity, and thus will likely influence how signs cluster together in our data. For example, in spoken language, a trend toward dispersion tends to also yield more divergence in form and meaning, due to the fact that “the dimensions available to create variation in the signal are limited to sequences of sounds, expressed in segmental and prosodic phonology” (Monaghan et al., 2014). However, in sign languages, dispersion does not inevitably lead to arbitrariness, because signs can be iconically mapped to referents along multiple dimensions and are not restricted to representations of sound properties of the referent (Sandler and Lillo-Martin, 2006). Additionally, because sign languages allow for simultaneous production of multiple dimensions of a sign, they are much less restricted in where distinctiveness appears in the wordform, while distinctiveness in spoken words is restricted temporally due to its sequential nature (Monaghan et al., 2014).

Our current analyses do not allow us to determine to what extent our semantic-phonological clusters are grouped on the basis of iconicity as opposed to systematicity. However, because the possibility of iconicity is dependent on both semantic domain and modality affordances as discussed above, we would expect clusters based on iconicity to have more constrained distribution relative to those based on systematicity. Moreover, although the presence of iconicity is more restricted, it is also more likely to pattern cross-linguistically. Because iconic wordforms make use of structural similarity and are mapped to meaning on the basis of real-world features of the referent, iconic patterns are more likely to be shared across languages, while systematicity is likely to be language-specific (Iwasaki et al., 2007; Gasser et al., 2011; Kantartzis et al., 2011; Monaghan et al., 2014; Dingemanse et al., 2015; Lockwood and Dingemanse, 2015). *Post-hoc* manual inspection of the clustering results can provide insight into the nature of these clusters and how they manifest across our two sign languages.

**Figure 8:**
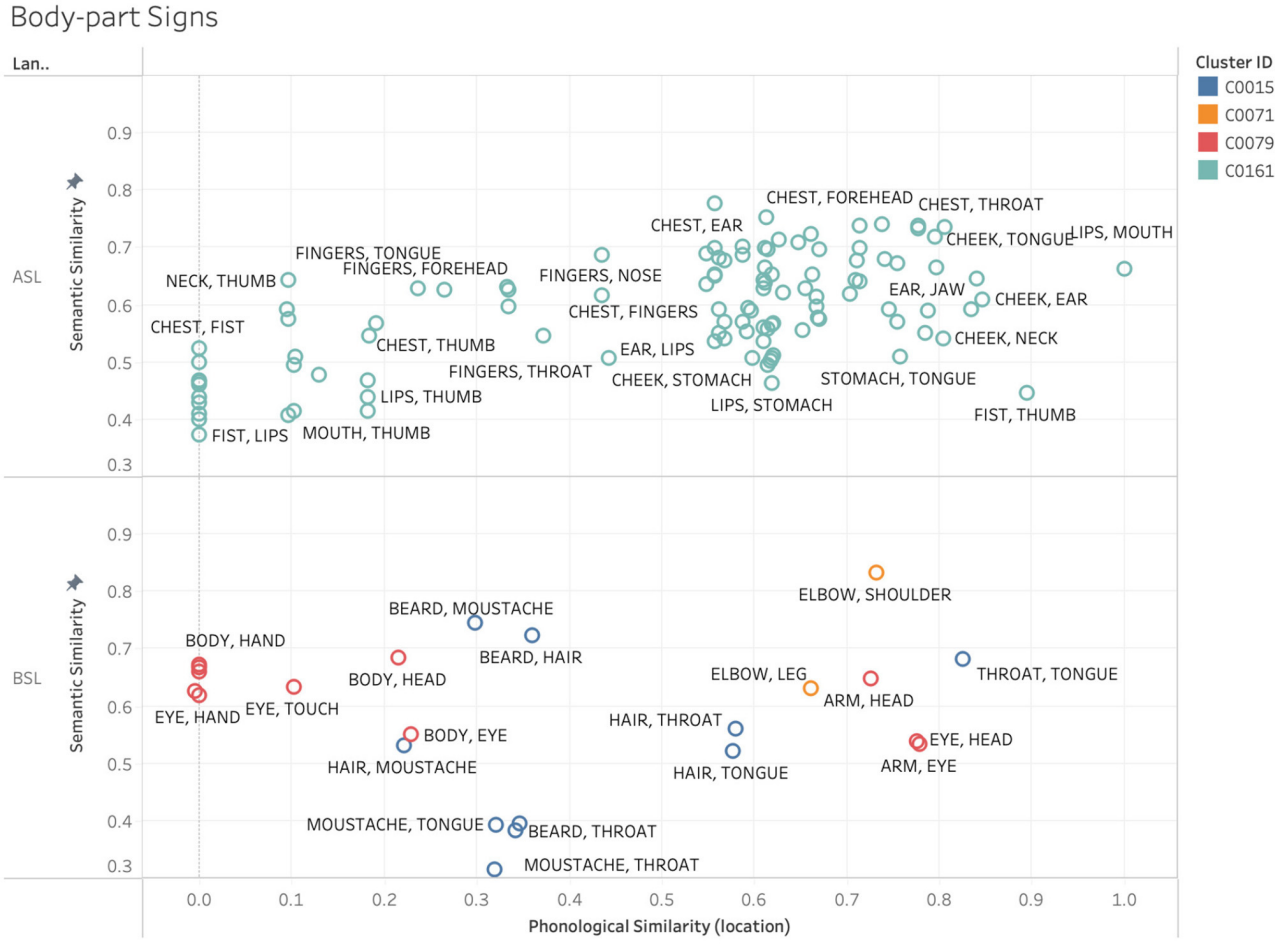
Example sign pairs relating to parts of the body in ASL and BSL.

As discussed above, certain types of meaning are more likely to be iconically realized than others, and in certain cases, this will be particularly facilitated in signed communication. For example, when considering how body parts are likely to be represented in a sign language, one might expect the default presence of the signer's own body in the visual space during communication to influence how various parts of the body are indicated. Locations on the body can be referenced *via* deixis, or pointing, without the need for any further abstraction. It is the affordances of the visuo-spatial modality that allow for this mapping between form and meaning for these concepts. However, we would only expect this location-based iconicity to give rise to phonological similarity between signs in cases where these locations are similar to each other. For signs, such as lips, teeth, tongue, and mouth, the iconic use of location should yield a high degree of phonological overlap. However, when we look at signs, such as head, hip, leg, and stomach, making use of location in this way should drive these signs apart phonologically. We would predict this location-based iconicity to be used in both ASL and BSL, as these affordances are not language specific, ultimately yielding a similar pattern regarding when iconicity should phonologically cluster these signs and when it should drive them apart. This ties into scholarship noting that there are particular locations in sign languages whose iconicity ties together particular families of signs that share similar meaning (Fernald and Napoli, 2000; van der Kooij, 2002). Figure 8 shows various sign pairs relating to parts of the body selected from a subset of clusters in the ASL and BSL datasets.

One obvious difference in the ASL and BSL data in Figure 8 is the number of data points, where the ASL data includes many more body-related signs than the BSL data. Additionally, the body-part signs all formed a single cluster in ASL, represented in the figure with the same cluster ID. The BSL body-part signs, in contrast, were distributed across three, indicated by three different colors on the plot. However, the strength of the phonological relationship, specifically in regards to location (*X*-axis), does appear to be largely dependent on the locations of the real-world referents for both ASL and BSL among this subset of signs. Note also that there exist constraints on the iconic use of location regarding body parts outside the signing space (e.g., feet) or taboo parts of the body (e.g., penis) which wouldn't use locative iconicity. Because of cases like these, we would not expect location to be used iconically across all body-part signs, thus weakening this correspondence.

**Figure 9:**
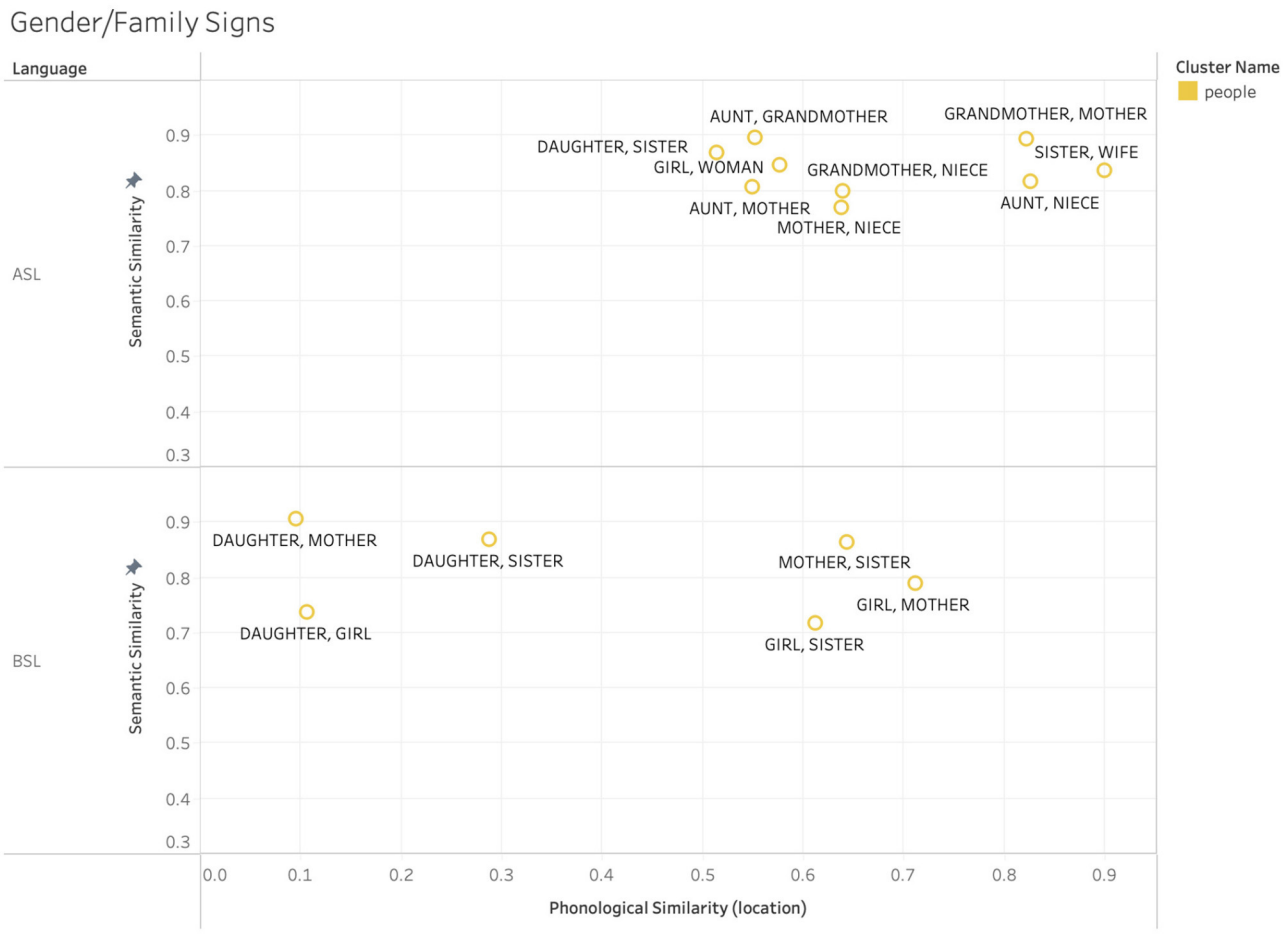
Example sign pairs relating to gender and family roles in ASL and BSL.

The patterns observed for body-part signs appear to be driven by location-based iconicity, and apply to both ASL and BSL. However, there are other non-arbitrary influences driving the clusters in our data that are systematic rather than iconic, and thus not likely to apply cross-linguistically. For example, in ASL many gendered signs such as those for family members adhere to a pattern wherein signs with female referents are articulated near the lower half of the head and signs with male referents are articulated near the upper half. While there may be iconic origins to the use of these locations, this pattern ultimately represents a non-arbitrariness that is systematic rather than iconic in contemporary ASL. Because of this, we would not expect this pattern to necessarily hold cross-linguistically, in much the same way that phonesthemes (e.g., the /gl-/ onset used in “glimmer,” “glow,” “gleam,” “glisten,” etc.) are often language specific. This point is exemplified in Figure 9, which shows a subset of female-gendered family signs from ASL and BSL clusters, specifically looking within phonological location. Within the ASL signs we see a higher degree of phonological overlap than within the BSL signs, with the ASL signs clustered to the right of the graph, while the BSL signs are distributed across a wider range. This exemplifies the expected pattern where a language specific schema that connects the meaning and form of a group of signs is reflected in the high degree of similarity between this cluster of forms in ASL, but not in BSL.

Taken together, the current findings contribute to our understanding of how and why non-arbitrariness is distributed across the linguistic systems of signed languages. Discussions of non-arbitrary relationships between form and meaning have been highlighted throughout much of the history of scholarship on sign languages, spanning not only discussions of iconicity, but also wider discussions of systematicities in form that rely on the affordances of the visual modality. Here, we used computational methodologies to contribute to this area of inquiry, using vector space models to quantify and examine patterns in the relationships between form and meaning in sign language lexicons. Our analyses suggest that the meaning of signs does, to some degree, contribute to the organization of their phonological properties. This relationship between form and meaning is most evident when we look at lexicons that are organized into more narrow semantic categories, with correspondences in phonological form being bounded by the semantic categories themselves. We see these relationships in both ASL and BSL, suggesting that these connections between the phonological form and the meaning of signs is not the property of just one sign language, but might be more generalizable, although the strength of this relationship may differ between languages.

However, the distribution of these non-arbitrary forms across the lexicon is mediated by several communicative and cognitive pressures. Not only do pressures toward phonological dispersion and clumsiness shape these trends, but so do various pressures from both real world referents as well as the constraints of the signing space. Certain pressures toward iconicity will likely influence the forms of signs across diverse sign languages, such as visual salience and affordances of the signing space, while other pressures would be expected to exist only within a given language or culture, such as gender pattern we see in ASL signs for people and family members. Additionally, the influence of iconicity does not always result in clustering signs together in phonological space. In many cases, signs that adopt similar iconic mappings to their referents are dispersed in the space, such as in the case of mouth and teeth versus mouth and leg. Expanding this analysis to larger datasets in future work will reveal further trends in the distribution and strength of these relationships.

Methodologically, the current work also contributes to a growing body of research on lexicon-wide computational analyses of sign languages, contributing a new way to approach the identification of form-meaning correspondences and their dispersion across the lexicon. Our analysis, similar to work like that of Thompson et al. (2020a), demonstrates that a vector space approach can be useful in modeling the semantic spaces of sign languages and we further show that VSMs can be harnessed to study patterns in non-arbitrary form-meaning correspondences for sign languages, even when using a relatively sparse representation of the lexicon. Because this computational approach enables the quantification of semantic and phonological similarity between many sign pairs, it is particularly useful for large scale, cross-linguistic comparisons of sign languages. We hope vector space approaches can be used in future work to further explore the pervasiveness of non-arbitrariness in different signed languages, expanding our understanding of the linguistic pressures that shape these systems.

## Data Availability Statement

The datasets presented in this study can be found in online repositories. The names of the repository/repositories and accession number(s) can be found at: https://github.com/EmreHakguder/SL_VSMS.

## Ethics Statement

Written informed consent was obtained from the individual(s) for the publication of any potentially identifiable images or data included in this article.

## Author Contributions

AM: contributed funding acquisition, data collection, writing, and draft preparation. CF and SK: contributed conceptualization, methodology, analysis, writing, and draft preparation. EH: contributed conceptualization, methodology, analysis, and writing. DB: contributed to the methodology, review-editing, and supervision. All authors contributed to the final version of the article and approved the submitted version.

## Funding

Funding for this project came from a grant awarded by the University of Chicago's *Center for Gesture, Sign, and Language*. This work was supported in part by NSF grant BCS 1918545.

## Conflict of Interest

The authors declare that the research was conducted in the absence of any commercial or financial relationships that could be construed as a potential conflict of interest.

## Publisher's Note

All claims expressed in this article are solely those of the authors and do not necessarily represent those of their affiliated organizations, or those of the publisher, the editors and the reviewers. Any product that may be evaluated in this article, or claim that may be made by its manufacturer, is not guaranteed or endorsed by the publisher.

## Footnotes

^1^We will be referring here to systematicity as it is used by Blasi et al. (2016) and Dingemanse et al. (2015) (among others), as a type of non-arbitrariness distinct from iconicity. Others such as Monaghan et al. (2014) label the whole of non-arbitrariness as “systematicity,” which is then divided into “absolute iconicity” and “relative iconicity” (Gasser et al., 2011). Under this framework, “relative iconicity” aligns with what we have referred to here as “systematicity.”

^2^This handshape may be derived from the emblem for “cuckold” or “cornuto” (horns) in Italian; however, neither hearing nor deaf Americans appear consider this resemblance as particularly iconic. This is evidenced by mock's very low iconicity rating (1.0 rating for non-signers and 1.7 rating for signers) on ASL-LEX (Sehyr et al., 2021).

^3^Iconicity as used here can also be thought of as “absolute iconicity” as described by Monaghan et al. (2011) and Gasser et al. (2011).

^4^As pointed out by Perlman et al. (2018), the claim that sign languages are overall more iconic has historically been based often on observation alone rather than empirical evidence. However, their cross-linguistic comparison of English, Spanish, ASL, and BSL found that “signs for particular meanings are fairly consistent in their level of iconicity in ASL and BSL, while there is greater variability between English and Spanish words…which may reflect that potential iconic mappings between form and meaning are more direct and transparent for many signs, and hence realized to a greater degree across different signed languages” (Perlman et al., 2018, p.8) and notes that “…this may indicate that signed languages are iconic in a qualitatively different–and, specifically, a more widely intuitive way—than spoken languages.” (Perlman et al., 2018, p.13).

^5^See Gibson et al. (2019) for an elaborated discussion of scholarship on the impact of the interaction between communicative pressures, including toward efficiency or toward retaining form-meaning mappings, on shaping linguistic systems.

^6^In Figure 2, each word is represented as a vector, and the two axes represent the coordinate basis of the vector space.

^7^Multi-syllabic compounds and signs with multiple distinct sequential morphemes were not included in the project datasets.

^8^GloVe is known to have advantage of reflecting both the local statistics and global statistics. It incorporates not only the local context information of words (as in the global matrix factorization methods (e.g., latent semantic analysis Deerwester et al., 1990) but also word co-occurrence, as in the local context window methods (e.g., the Word2vec model Mikolov et al., 2013).

^9^For example, the BSL sign cuddle is assigned with categorical labels for each feature (i-a) in the annotated phonology data. Once one-hot-encoded, the sign can be represented as a vector expressed with 0 and 1 values for each possible phonological feature (i-b). Once dimensionality reduction is applied to the one-hot-encoded representation of cuddle, the sign is represented in a vector of a lower dimension with real numbers as in (i-c). The vector in (i-c) is the actual vector representation of cuddle we used for calculating phonological similarity between signs. The vector size and numeric values for each sign varies by parameter (i.e., handshape, location, movement). More than 96% of the original information was retained after dimensionality reduction for all parameters.

(i) cuddle
a. Annotated with labels for each feature: {Spread: “unspread,” Joint Configuration: “flexed,” SelectedFingers: “all”…}
b. One-hot-encoded sign: [Spread_spread: 0, Spread_unspread: 1, Joint_Configuration_curved: 0, Joint_Configuration_stacked: 0, Joint_Configuration_flexed: 1, SelectedFingers_ring: 0, SelectedFingers_middle: 0, SelectedFingers_all: 1…]
c. Final vector representation output after dimensionality reduction: [1.8510536316565696, 1.5249889622005965, 0.629900379775755,…, 0.003631133800598263].
